# A Role for Taiman in Insect Metamorphosis

**DOI:** 10.1371/journal.pgen.1004769

**Published:** 2014-10-30

**Authors:** Jesus Lozano, Takumi Kayukawa, Tetsuro Shinoda, Xavier Belles

**Affiliations:** 1 Institut de Biologia Evolutiva (CSIC-UPF), Barcelona, Spain; 2 Division of Insect Sciences, National Institute of Agrobiological Sciences, Tsukuba, Ibaraki, Japan; Howard Hughes Medical Institute, United States of America

## Abstract

Recent studies *in vitro* have reported that the Methoprene-tolerant (Met) and Taiman (Tai) complex is the functional receptor of juvenile hormone (JH). Experiments *in vivo* of Met depletion have confirmed this factor's role in JH signal transduction, however, there is no equivalent data regarding Tai because its depletion in larval or nymphal stages of the beetle *Tribolium castaneum* and the bug *Pyrrhocoris apterus* results in 100% mortality. We have discovered that the cockroach *Blattella germanica* possesses four Tai isoforms resulting from the combination of two indels in the C-terminal region of the sequence. The presence of one equivalent indel-1 in Tai sequences in *T. castaneum* and other species suggests that Tai isoforms may be common in insects. Concomitant depletion of all four Tai isoforms in *B. germanica* resulted in 100% mortality, but when only the insertion 1 (IN-1) isoforms were depleted, mortality was significantly reduced and about half of the specimens experienced precocious adult development. This shows that Tai isoforms containing IN-1 are involved in transducing the JH signal that represses metamorphosis. Reporter assays indicated that both *T. castaneum* Tai isoforms, one that contains the IN-1 and another that does not (DEL-1) activated a JH response element (*k*JHRE) in *Krüppel homolog 1* in conjunction with Met and JH. The results indicate that Tai is involved in the molecular mechanisms that repress metamorphosis, at least in *B. germanica*, and highlight the importance of distinguishing Tai isoforms when studying the functions of this transcription factor in development and other processes.

## Author Summary

Insect metamorphosis is one of the most fascinating processes of animal development. However, the mechanisms governing metamorphosis only started to be unveiled in the last century, when physiological research revealed that the main factor involved is juvenile hormone (JH), which represses metamorphosis in juvenile stages. Further steps to elucidate the molecular mechanisms underlying the action of JH remained elusive until recently, when the transcription factor Methoprene-tolerant (Met) was reported to be the JH receptor in the context of metamorphosis. Further experiments *in vitro* suggested that Met did not act alone as the JH receptor, but had to heterodimerise with another protein, Taiman (Tai). Unfortunately, Tai depletion experiments to demonstrate this protein's involvement in metamorphosis proved unsuccessful because they resulted in 100% mortality. We have discovered that Tai is expressed in a number of isoforms, and selective depletion has shown that at least some of these are involved in transducing the JH signal that represses metamorphosis. Moreover, our results show that the whole range of isoforms should be considered when studying Tai functions.

## Introduction

Insect metamorphosis, either through gradual morphogenesis (hemimetaboly) or abrupt morphogenesis mediated by the pupal stage (holometaboly), is essentially regulated by two hormones, 20-hydroxyecdysone (20E) and juvenile hormone (JH). 20E triggers the successive moults throughout the life cycle, whereas JH represses metamorphosis [1]–[3]. A great deal of information has been obtained on the 20E signalling pathway and corresponding transcription factors, many of which belong to the nuclear receptor superfamily [4], [5]. Conversely, the molecular mechanisms underlying the action of JH remained enigmatic until recently, when the transcription factor Methoprene-tolerant (Met) was reported as the JH receptor [6], and a number of components of the JH signalling pathway were identified [7], [8].

Met is a member of the basic-helix-loop-helix (bHLH)/Per-Arnt-Sim (PAS) family of transcription factors that was discovered in 1986 as a factor determining resistance towards the toxic effects of the JH analogue methoprene in some *Drosophila melanogaster* strains [9], [10]. Years later, Miura et al. [11] showed that the *D. melanogaster* Met protein could bind JH with a very high affinity (Kd of 5.3 nM), providing the first hint that Met might play a role in JH reception. More recently, it has been observed that depleting Met mRNA levels with RNAi in early larval stages of the holometabolan species *Tribolium castaneum* triggers precocious pupal morphogenesis [12], [13], showing that Met is involved in the transduction of the anti-metamorphic JH signal. RNAi studies have also demonstrated the JH-transducing role of Met in the metamorphosis of the bug *Pyrrhocoris apterus* [14] and the cockroach *Blattella germanica* [15], both hemimetabolan species. Using Met from *T. castaneum*, Charles et al. [6] confirmed that the JH binding affinity is high (Kd of 2.9 nM in this case), that the PAS-B motif of Met is required and is sufficient to bind JH, and that when JH binds to a Met moiety in a Met-Met homodimer, the homodimer dissociates and JH+Met binds to another bHLH-PAS transcription factor named Taiman (Tai).

Tai was originally discovered in *D. melanogaster* when searching for factors regulating border cell migration during oogenesis [16]. Later, Zhu et al. [17] used a yeast two-hybrid system to identify proteins interacting with the transcription factor βFtz-F1, a nuclear receptor associated with ecdysone signalling in the fat body of the mosquito *Aedes aegypti*, and they recovered a Tai homolog which they called FISC (βFtz-F1 Interacting Steroid receptor Coactivator). In a similar yeast two-hybrid screening, Tai/FISC was again found in *A. aegypti* [18] as well as in *T. castaneum* [19], this time interacting with Met. To further complicate the nomenclature, the Tai homolog of *T. castaneum* was named SRC due to its homology with mammal SRCs.

It was subsequently found that downstream of Met, the JH signal is transduced by Krüppel-homolog 1 (Kr-h1), a transcription factor with a DNA binding domain of eight zinc fingers. The function of Kr-h1 as a transducer of the anti-metamorphic action of JH was first demonstrated by ectopic expression in *D. melanogaster* [20]. Soon after, RNAi experiments in holometabolan species like *T. castaneum* [21], as well as hemimetabolan species, such as *B. germanica* [22], *P. apterus* and *Rhodnius prolixus* [14], revealed that depleting Kr-h1 mRNA levels in early juvenile stages triggered precocious metamorphosis in all cases.

While there is robust data available showing that Met plays a role in JH reception in metamorphosis and that Kr-h1 transduces the anti-metamorphic JH signal downstream of Met, the possible role of Tai in this process has remained unclear. Using approaches *in vitro*, Tai-dependent expression of Kr-h1 has been reported in the Aag-2 line of *A. aegypti* cells [19], in NIAS-Bm-aff3 cells of the silkworm *Bombyx mori* [23] and in Tc81 cells of *T. castaneum* [24]. The results obtained by Kakuyawa et al. [23] in NIAS-Bm-aff3 cells of *B. mori* are relevant because they demonstrated the occurrence of a new JH response element, *k*JHRE, in the promoter region of *B. mori* Kr-h1that contains an E-box to which bHLH-PAS proteins may bind. Reporter assays in mammalian HEK293 cells, which presumably lack JH signalling elements, showed that *k*JHRE-specific reporter activity was weakly activated in the presence of JH when the complete open reading frame (ORF) of *B. mori* Met was expressed, although reporter activity significantly increased when Tai ORF was coexpressed with Met. The results therefore suggested that Met and Tai jointly interacted with the *k*JHRE found in the Kr-h1 promoter and that this interaction was JH-dependent [23]. Equivalent work carried out on the new Tc81 embryonic cell line of *T. castaneum* [24] allowed the identification and functional characterisation of a *k*JHRE, found in the promoter region and the first intron of *T. castaneum* Kr-h1, which was identical to that reported in *B. mori*. Experiments with mammalian HEK293 cells and *Drosophila* S2 cells showed that *k*JHRE reporter activity was weakly activated when the complete ORF of *T. castaneum* Met was expressed in the presence of JH, but when Tai ORF was coexpressed with Met, reporter activity increased [24].

Data demonstrating the involvement of Tai in metamorphosis is practically non-existent for approaches *in vivo*, not because Tai depletion experiments have been not attempted, but because these experiments, carried out on *T. castaneum* larvae [25] and *P. apterus* nymphs [26], resulted in 100% mortality. The only information *in vivo* derives from assays carried out on *T. castaneum* larvae which showed that treatment with hydroprene (a JH analogue) stimulates Kr-h1 expression, whereas depletion of Tai overrides this stimulatory effect [19]. The paradox is that current models explaining the mechanisms repressing insect metamorphosis consider Tai to be the partner of Met in the JH heterodimer receptor at the top of the repressor pathway [1], [2], [7], [8], but, unfortunately, no convincing evidence *in vivo* of Tai's contribution to metamorphosis has been forthcoming. The results reported herein show that Tai is involved in repressing metamorphosis. The path to this conclusion involved working with the cockroach *B. germanica*, which is an especially favourable model for carrying out functional RNAi experiments, and in which different isoforms of Tai can be examined.

## Results

### *B. germanica* has alternative splicing isoforms of Tai

**Figure 1:**
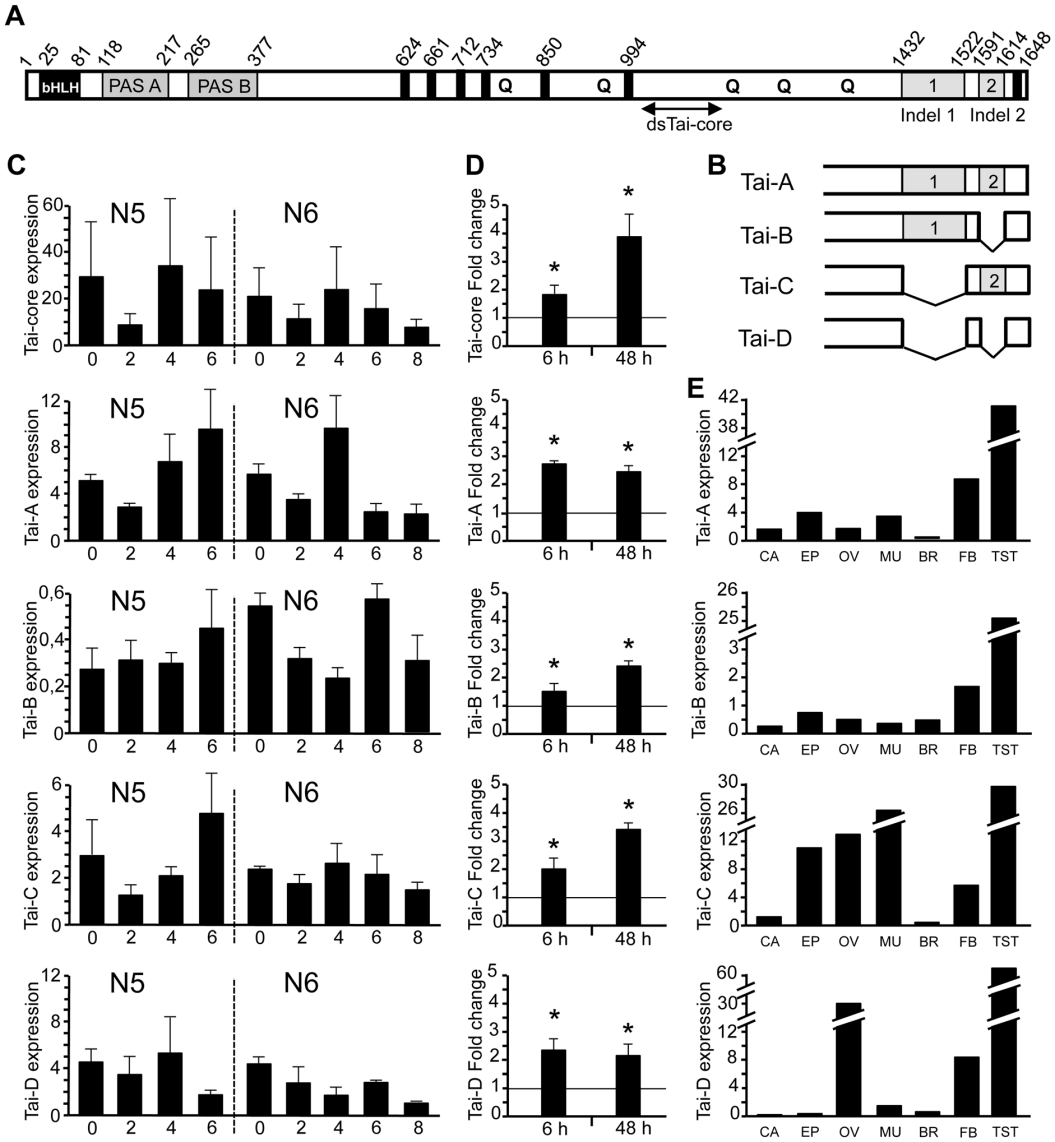
Structure and expression of BgTai and their isoforms in *Blattella germanica*. (A) Organization of BgTai protein in different domains; in addition to the bHLH region and the two PAS domains, it contains seven LxxLL motifs indicates as black bars, five regions rich in Glutamine, indicated with “Q”, and two Insertions/Deletions towards the 3′ region one (IN-1) of 276 bp and the other (IN-2) of 74 bp; also indicated is the region were the dsTai-core used in RNAi studies was designed. (B) Scheme representing the C-terminal region of the four isoforms of BgTai. (C) Expression of BgTai mRNA in whole body of female nymphs in penultimate (N5) and last (N6) instar; from top to down it is showed the expression of the ensemble of isoforms (amplified with primers designed to regions common to all them), and that of each isoform A, B, C and D (amplified with respective specific primers). (D) Effect of JH III treatment (20 µg) on BgTai expression, on the ensemble of isoforms, and specifically on each one; JH was topically applied in freshly emerged N6, and BgMet mRNA levels were measured 6 and 48 h later. (E) Expression of each BgTai isoform in different tissues of females in N6D0: *corpora allata* (CA), epidermis of the thoracic discs (EP), ovaries (O), muscle (M), brain (B), fat body (FB), and in testicles (TST) from males of the same age. Each point in C and D represents 4 biological replicates and results are expressed as the mean ± SEM, whereas those in E represent a pool of 5 specimens; data in C and E are expressed as copies of BgTai mRNA per 1000 copies of BgActin-5c mRNA; data in D are normalized against the dsMock-treated samples (reference value = 1), and the asterisk indicates statistically significant differences with respect to controls (p<0.05), according to the REST software tool [38].

BLAST search of Tai in transcriptomes of *B. germanica* followed by PCR amplifications gave an ORF sequence of 4914 bp whose conceptual translation rendered a 1638 amino acid protein with sequence similarity to insect Tai/FISC/SRC proteins and that we called BgTai. The sequence contained the bHLH, PAS A and PAS B domains, as well as seven LxxLL motifs and several glutamine-rich regions characteristics of Tai/FISC/SRC proteins (Figure 1A), and top BLAST scores were obtained from Tai/FISC/SRC orthologs of other insects. The phylogenetic analysis of vertebrate SRC and insect Tai/FISC/SRC shows that insect proteins cluster a part, as a sister group of the vertebrate SRCs that is formed by three separated nodes corresponding to SRC-1, SRC-2 and SRC-3 subgroups (Figure S1). Therefore, it seems appropriate to use a single name, Taiman, for all insect representatives of the SRC superfamily of proteins. BgTai protein shows the highest amino acid identity with the Tai ortholog of the hemimetabolan species *Pediculus humanus*, both in the overall protein sequence and in the bHLH domain (57 and 82% identity, respectively); identity values decreased for *D. melanogaster* (25 and 49%) and *A. aegypti* (32 and 51%) (Figure S2).

Four different transcripts of BgTai corresponding to four variants of the protein were amplified from RNA extracts obtained from last instar nymphs. The four variants result from the combination of two indels localized towards the C-terminal region of the sequence. The first insertion (IN-1) starts at nucleotide 4293 from the first Met triplet and has a length of 276 nucleotides, whereas the second (IN-2) starts at nucleotide 4771 and is 74 nucleotides long. The deletion corresponding to IN-2 (DEL-2) produces a frame-shift and an early in-frame stop codon that slightly truncates the protein in the C-terminal region. We named the four variants as follows: BgTai-A (which has IN-1 and IN-2, GenBank accession number HG965205), BgTai-B (has only IN-1, GenBank accession number HG965206), BgTai-C (has only IN-2, GenBank accession number HG965207), and BgTai-D (has neither IN-1 nor IN-2, GenBank accession number HG965208) (Figure 1B). The common regions of the four BgTai variants have identical nucleotide sequence, which suggests that they are alternative splicing products of the same gene.

According to public database information, an indel-1 equivalent to that found in BgTai occurs in the Tai sequences of *T. castaneum*, *D. melanogaster* and the honey bee *Apis mellifera* (Figure S3). In the case of *B. mori*, the available Tai sequence reported by Kayukawa et al. [23] is likely to be a DEL-1 isoform. Using *T. castaneum* specific primers and adult whole body cDNA of *T. castaneum* as a template, we amplified the region around the indel-1 and we obtained two sequences, one containing the insertion and the other without it (Figure S4), thus confirming that both IN-1 and DEL-1 variants exist in this species.

### Tai isoforms are expressed with slightly different patterns during the last nymphal instars of *B. germanica* and expression is unevenly distributed among tissues

We firstly studied the expression of BgTai in fifth (penultimate, N5) and sixth (last, N6) nymphal instars using primers designed in a region common to the four BgTai isoforms, which gave the expression pattern of the four-isoform ensemble. Values fluctuate between 10 and 30 mRNA copies per 1000 copies of actin mRNA (Figure 1C). Using isoform-specific primer pairs, we determined the expression patterns of each isoform to reveal that BgTai-A is the most abundantly expressed, followed by BgTai-C, BgTai-D and BgTai-B. The pattern of BgTai-A was similar to that of the four-isoform ensemble (denoted as “Tai-core” in Figure 1C), but the other isoforms' patterns showed slight differences amongst themselves (Figure 1C). We then determined that BgTai expression is up-regulated upon JH treatment and that the stimulatory effect is induced in all isoforms and, in general, increases with time (Figure 1D). Expression studies in different tissues revealed that all isoforms are abundantly expressed in testes; clear differential expression of the different isoforms was observed in ovaries (BgTai-D being the most expressed) and muscle (where BgTai-C is the most expressed) (Figure 1E).

### Depletion of all Tai isoforms has lethal effects

**Figure 2:**
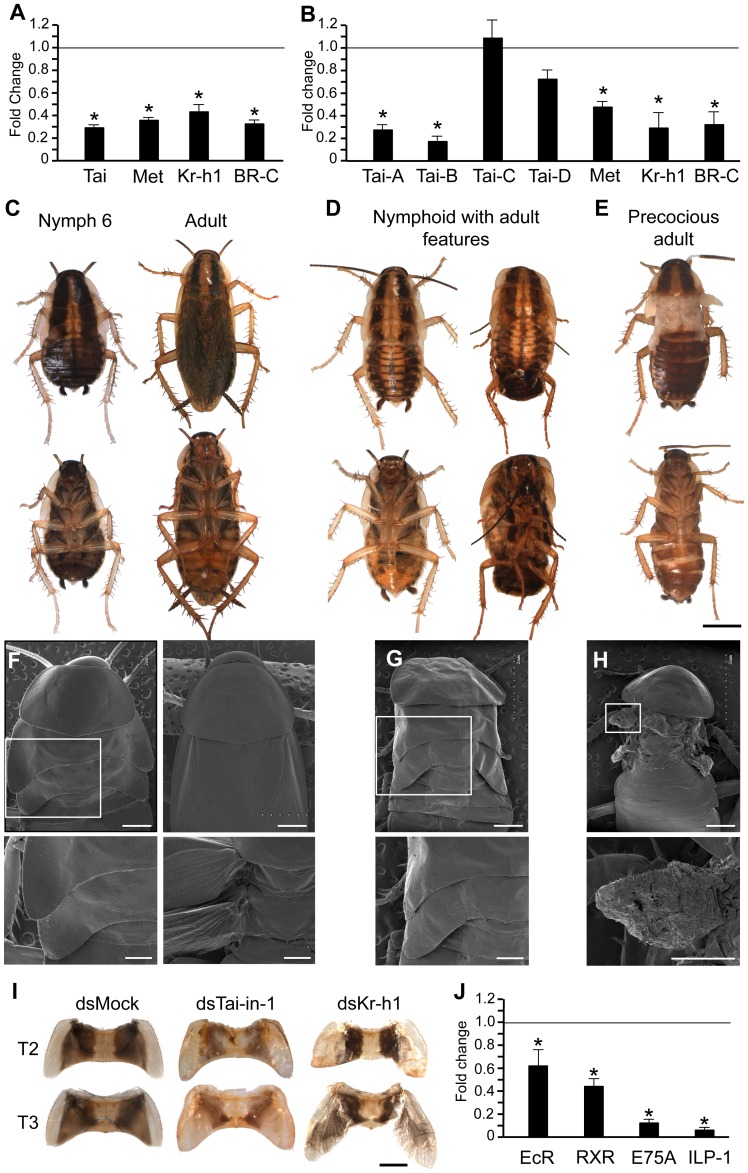
Function of BgTai in *Blattella germanica* metamorphosis. (A) Effects, at transcript level, of dsTai-core treatment in N5; N5 females received two 3-µg doses of dsTai-core, one on N5D0 and the other on N5D3, and transcript levels (of Tai, Met, Kr-h1 and BR-C) were measured on N5D6; controls received an equivalent treatment with dsMock. (B) Effects, at transcript level, of dsTai-in-1 treatment in N4; the experimental design was equivalent to that used in N5, with a double treatment, one on N4D0 and the other on N4D3; transcript levels (of Tai-A, Tai-B, Tai-C, Tai-D, Met, Kr-h1 and BR-C) were measured on N5D6. (C–E) Dorsal and ventral view of specimens resulting from dsTai-in-1 treatment in N4; normal last nymphal instar and adult obtained from dsMock treatments (C); nymphoids with adult features obtained (instead of N6) from dsTai-in-1 treatments (D); precocious adult obtained (instead of N6) from dsTai-in-1 treatments (E). (F–H) SEM images of T2 and T3 (general and detail of the lateral parts) from specimens resulting from dsTai-in-1 treatment in N4; normal last nymphal instar and adult obtained from dsMock treatments (F); nymphoid of the figure D, right (G); precocious adult from the figure E (H). (I) Dissected T2 and T3 of: N6 control (dsMock-treated), a nymphoid with adult features obtained after dsTai-in-1 treatment, a nymphoid with adult features obtained after dsKr-h1 treatment. (J) Effects of dsTai-in-1 treatment on N4 on the expression of EcR, RXR, E75A and ILP-1 measured on N5D6. Each point of quantitative data in histograms A, B and J represents 4 biological replicates and results are expressed as the mean ± SEM; data are normalized against the dsMock-treated samples (reference value = 1), and the asterisk indicates statistically significant differences with respect to controls (p<0.05), according to the REST software tool [38]. Scale bars in C, D, E = 3 mm, in F, G, H = upper and bottom respectively = 1 and = 0.5 mm, and in I = 1 mm.

To test whether BgTai has a role in *B. germanica* metamorphosis, we designed a dsRNA based on a region common to all isoforms (dsTai-core, Figure 1A) in order to deplete the four-isoform ensemble. Firstly, we injected two 3-µg doses of dsTai-core into N5 females, one when freshly emerged (N5D0) and the other on day 3 (N5D3). Controls were equivalently treated with dsMock. Transcript measurements carried out on N5D6 indicated that the whole ensemble of Tai mRNAs was significantly down-regulated (*ca.* 70%) in dsTai-core-treated specimens in comparison with controls (Figure 2A). In these specimens, mRNA levels of Met, Kr-h1 and Broad complex (BR-C, an ecdysone- and JH-dependent transcription factor that promotes wing primordia growth in *B. germanica* [27] were also significantly reduced (*ca.* 70, 65 and 70%, respectively) (Figure 2A).

At the phenotypic level, the dsMock group (n = 14) moulted to N6 and then to normal adults, but specimens treated with dsTai-core (n = 18) died within the same instar (Table S1). These nymphs showed reduced motility, stopped growing, became darker (Figure S5A) and finally died 7–10 days after the last treatment. Equivalent experiments carried out on N4, injecting two 3-µg doses of dsTai-core in N4D0 and in N4D3 (n = 12), produced 100% mortality, while equivalently dsMock-treated specimens (n = 10) developed normally until the adult stage (Table S1). To reduce mortality, we administered a single 0.5-µg dose of dsTai-core in N4D0 (n = 7) or in N5D0 (n = 16), which still provoked almost 100% mortality before reaching N6 (Table S1). Treatments in N4D0 with a single 0.3-µg dose (n = 16) reduced nymphal mortality to *ca.* 40% but the survivors moulted to normal adults, in few cases with slightly wrinkled wings. Finally, treatments with 0.2 µg of dsTai-core in N4D0 (n = 12) did not trigger mortality and all specimens moulted to adults, in most cases (*ca.* 70%) with the wings wrinkled (results summarised in Table S1); intriguingly, when they were in N6 the lateral expansions of the mesothorax (T2) and metathorax (T3) looked somewhat longer and more transparent than normal (Figure S5B), however they all finally moulted to adults.

### Depletion of BgTai isoforms containing the insertion 1 is not lethal and triggers precocious formation of adult features

Considering the possibility that each Tai isoform assumes different specific roles, we designed a dsRNA based on the 276 nucleotides of insertion 1 (dsTai-in-1), which should specifically deplete the two longer isoforms, Tai-A and Tai-B (Figure 1B). We injected two 3-µg doses of dsTai-in-1 in N4 females, one in N4D0 and the other in N4D3. Controls received an equivalent treatment with dsMock. Transcript measurements carried out on N5D6 showed that the treatment specifically targeted the long isoforms Tai-A and Tai-B, which were effectively depleted (*ca.* 75 and 85%, respectively), whereas Tai-C and Tai-D transcripts were unaffected (Figure 2B). dsTai-in-1-treatment resulted in a significant reduction of Met, Kr-h1 and BR-C mRNA levels (*ca.* 50, 70 and 65%, respectively) (Figure 2B).

At the phenotypic level, only 5 out of the 27 (19%) specimens treated with dsTai-in-1died before N6. A total of 19 out of the 22 survivors (86%), instead of moulting to normal N6, moulted to nymphoids with adult features such as a general yellowish colouration and enlarged lateral expansions in T2 and T3 that appeared flexible in the distal part. About 40% of these 19 specimens completed the ecdysis (Figure 2D, left) and remained in this stage for three to six weeks without moulting again, whereas the other ∼60% did not complete the ecdysis, thus parts of the exuvium remained attached to the abdomen (Figure 2D, right); these specimens died within 3–4 days after attempting ecdysis. Finally, 3 out of the initial 22 survivors (14%) underwent apolysis to N6 but did not start ecdysis. Manual removal of the exuvium revealed that they had the general shape and colouration of a precocious adult, showing wing-like although heavily folded structures at both sides of T2 and T3 (Figure 2E, Table S1).

Scanning electron microscopy examination of T2 and T3 in dsMock-treated specimens (Figure 2F), compared with dsTai-in-1-treated specimens (Figures 2G and H), revealed more details of the enlarged and partially flexible lateral expansions of the “nymphoids with adult features” (Figure 2G) and the wing-like structures articulated to the pleura of the specimens categorised as “precocious adults” (Figure 2H). The wing-like structures of these precocious adults are reminiscent of the heavily folded wings forming within the wing pocket of T2 and T3 lateral expansions in last instar nymphs when observed just before the imaginal ecdysis (Figure S6). The features of T2 and T3 lateral expansions of the nymphoids with adult features obtained after dsTai-in-1 treatment (Figure 2I) are similar to those observed in nymph-adult intermediates obtained by depleting Kr-h1 with a single dsRNA treatment, as described by Lozano and Belles [22].

The recurrent moulting problems and wing size deficiencies of the specimens treated with dsTai-in-1 led us to measure transcript levels of genes related to the ecdysone signalling pathway, such as EcR, RXR and E75A, as well as those of ILP-1. In all genes, mRNA levels were significantly lower than those of the controls, especially in the case of E75A and ILP-1 (Figure 2J).

### Depletion of BgTai isoforms containing the insertion 2

**Figure 3:**
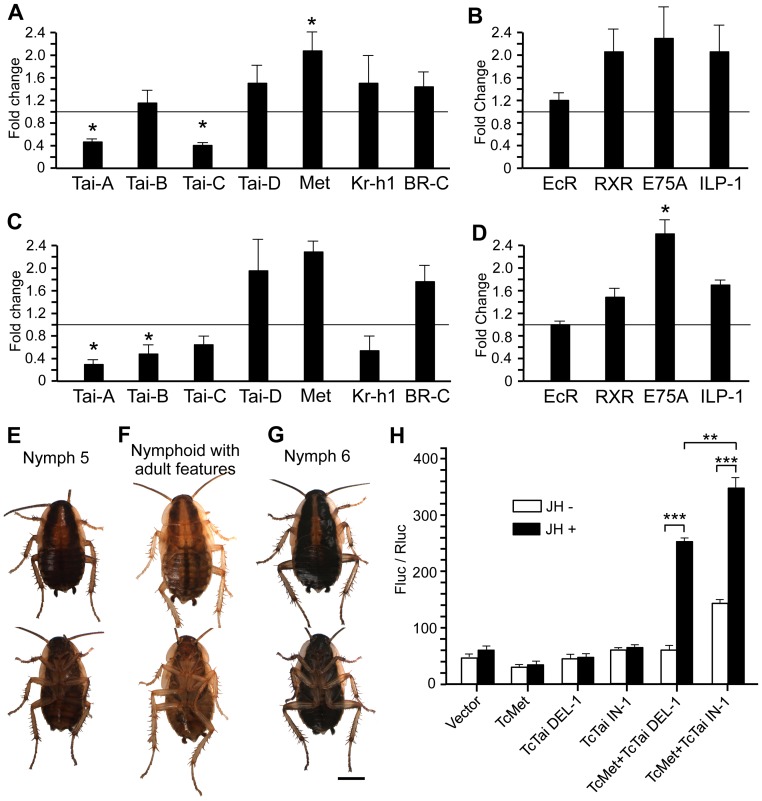
Functional study of IN-2-containing isoforms of BgTai and reporter assays to assess the contribution of IN-1 of TbTai to transduce the JH signal. (A) Effects, at transcript level, of dsTai-in-2 treatment in N4; N4 females received two 3-µg doses of dsTai-in-2, one on N4D0 and the other on N4D3, and transcript levels (of Tai-A, Tai-B, Tai-C, Tai-D, Met, Kr-h1 and BR-C) were measured on N5D6; controls received an equivalent treatment with dsMock. (B) Effects of the dsTai-in-2 treatment on the expression of EcR, RXR, E75A and ILP-1 measured also on N5D6. (C) Effects, at transcript level, of dsTai-in-1 plus dsTai-2 treatment in N4; specimens received an injection of dsTai-in-1 (3 µg) plus dsTai-in-2 (3 µg) in N4D0, and the treatment was repeated in N4D3; controls received an equivalent treatment with dsMock. (D) Effects of the dsTai-in-1 plus dsTai-in-2 treatment on the expression of EcR, RXR, E75A and ILP-1 measured also on N5D6. (E–G) Dorsal and ventral view of specimens resulting from dsTai-in-1 plus dsTai-in-2 treatment; normal N5 obtained from this or from dsMock treatments (E); nymphoids with adult features obtained (instead of N6) from dsTai-in-1 plus dsTai-in-2 treatments (F); normal N6 obtained from dsMock treatments (G). Each point of quantitative data in histograms A to D represents 4 biological replicates and results are expressed as the mean ± SEM; data are normalized against the dsMock-treated samples (reference value = 1), and the asterisk indicates statistically significant differences with respect to controls (p<0.05), according to the REST software tool [38]. Scale bars in E–G = 3 mm. (H) Reporter assays to study the JH-dependent interaction of TcMet and TcTai DEL-1 or TcTai IN-1 with *k*JHRE in *Drosophila* S2 cells. The cells were transfected with a *k*JHRE-reporter vector (−477 to +1883, pGL4.14), a reference reporter plasmid carrying *Renilla* luciferase (pIZT-Rluc) and a plasmid expressing the full ORF of TcMet and/or TcTai IN-1 or TcTai DEL-1. Cells were treated with 1 µM of JH III for 24 h. Reporter activity was measured using a Dual-Luciferase Reporter Assay System. Each bar indicates the mean ± SEM (n = 6). The asterisks indicate statistically significant differences between measurements (***p<0.001; **p<0.01) using a two-tailed t-test (n = 6).

As a next step, we attempted the depletion of the isoforms containing the insertion 2, using a dsRNA encompassing the 74 nucleotides of this insertion (dsTai-in-2). This dsRNA should specifically deplete the isoforms Tai-A and Tai-C (Figure 1B). We injected two 3-µg doses of dsTai-in-2 in N4 females, one in N4D0 and the other in N4D3, whereas controls received an equivalent treatment with dsMock. On N5D6, transcript measurements indicated that dsTai-in-2 targeted the isoforms Tai-A and Tai-C, as expected, which resulted in a rather modest depletion (approximately 55 and 60% for Tai-A and Tai-C, respectively), whereas Tai-B and Tai-D transcripts were unaffected (Figure 3A). These treatments with dsTai-in-2 did not reduce the mRNA levels of Met, Kr-h1 and BR-C (Figure 3A). In the same samples, we also measured mRNA levels of the ecdysone pathway genes (EcR, RXR and E75A), as well as those of ILP-1, and also in these genes, dsTai-in-2-treatment did not reduce transcript levels; only Kr-h1 showed somewhat lower average levels, although differences with respect to controls were not statistically significant (Figure 3B).

A total of 33 dsTai-in-2-treated specimens were left alive, and 3 of them (9%) died in N4, 5 (15%) in N5, 4 (12%) in N6 and 4 (12%) during the adult molt. A total of 17 (52%) survived during all the experiment and molted to normal adults (Table S1). In all cases, the morphology of N5, N6 and adult of these specimens was identical to that of controls, and they did not experience ecdysis problems, neither in the nymphal nor in the imaginal molts. These results, however, should be considered with caution due the modest depletion of Tai-A and Tai-C reached in dsTai-in-2-treated specimens.

Finally, we depleted the isoforms containing both insertions 1 and 2, by injecting dsTai-in-1 (3 µg) and dsTai-in-2 (3 µg) in N4D0 in a single injection, and repeating the treatment in N4D3. These treatments should target Tai-A, Tai-B and Tai-C isoforms (Figure 1B). Controls received two 6-µg doses of dsMock, one in N4D0 and the other in N4D3. Transcript measurements performed on N5D6, indicated that treatment with dsTai-in-1 plus dsTai-in-2 significantly reduced transcript levels of Tai-A (*ca.* 70%) and Tai-B (*ca.* 50%), and tended to reduce (*ca.* 40%) those of Tai-C. Tai-D transcripts were unaffected (Figure 3C). Transcript levels of Met and BR-C were higher with respect to controls, whereas those of Kr-h1 tended to be lower (Figure 3C). Transcript levels of EcR were not modified, and those of RXR, E75A and ILP-1tended to be higher than in controls (Figure 3D).

With respect to the phenotype, 7 out of the 20 specimens treated with dsTai-in-1 plus dsTai-in-2 died shortly after the second injection. The remaining 13 specimens molted to N5 normally (Figure 3E) and then moulted to nymphoids with adult features like a general yellowish colouration and enlarged and more or less flexible lateral expansions in T2 and T3 (Figure 3F, Table S1). The morphology of these specimens reminded that of the 86% of nymphoids with adult features obtained in the treatments with dsTai-in-1 (cf. Figure 2D). These 13 specimens completed the ecdysis normally and remained in this N6 nymphoid stage for three to four weeks without moulting again and then died. The dsMock-treated controls (n = 15) moulted to N5 and then to N6 (Figure 3G) and to adult normally.

### Transduction of the JH signal of *Tribolium* Tai isoforms *in vitro*

We then wondered about possible differences of signal transduction efficiency in different Tai isoforms. Given the availability of tools for JH signalling analysis in *T. castaneum* [24], these mechanistic studies were conducted using the transcription factors of this species. There is no indel-2 equivalent in the two known *T. castaneum* sequences of Tai (TcTai), but there is a homologous indel 1. Therefore, we carried out the reporter assays in *Drosophila* S2 cells, using a kJHRE reporter vector [24] combined with TcMet and TcTai DEL-1 or TcTai IN-1, in the presence or absence of JH.

Results (Figure 3H) showed that the wild type reporter did not elicit any JH-dependent induction in cells whether neither TcMet nor TcTai DEL-1 or TcTai IN-1 were expressed, or in cells where only one construct, either TcMet, or TcTai DEL-1 or TcTai IN-1 was expressed, as expected. Conversely, significant JH-dependent induction was detected in cells in which both, TcMet plus TcTai DEL-1 or TcMet plus TcTai IN-1 were expressed. Comparing the results in the presence of JH, the combination TcMet plus TcTai IN-1 elicited a significantly higher response with respect to that of TcMet plus TcTai DEL-1 (Figure 3H). However, and intriguingly, TcTai IN-1 also increased a basal, JH-independent reporter activity, thus the specific activation by JH became reduced. Comparing JH-dependent activation relative to activity without JH, and separately for each set of transfected proteins, it can be estimated that TcTai DEL-1 mediates a higher activation than Tai IN-1 in response to JH. Therefore, both Tai isoforms are functional in transducing the JH signal, although TcTai DEL-1 appears to elicit a somewhat higher JH-specific response than the TcTai IN-1.

## Discussion

### Tai isoforms

We have found a Tai homologue in *B. germanica*, as shown by sequence comparisons and phylogenetic analysis. The latter included not only insect sequences but also vertebrate homologues (SRC sequences, some of which are also known by the names AIB1, ACTR, RAC3, TRAM-1 and p160/NCoA-1) and showed that insect Tai sequences (some of which are also known by the names FISC, SRC, NRC3 and NcoA) cluster a part, as a sister group of the vertebrate SRC cluster. Considering the above, we propose using the name Taiman (Tai) for all insect representatives of the SRC superfamily of proteins, given that they cluster as a well differentiated monophyletic group within the SRC superfamily tree and because Taiman was the first name coined for an insect SRC [16].

Previous studies on insect Tai homologues, whether in *D. melanogaster* [16], *A. aegypti* [17], [18], *T. castaneum* [19] or *P. apterus* [26], did not provide evidence of isoforms in the C-terminal region of the molecule. In *B. germanica*, we report four isoforms resulting from the combination of two indels in the C-terminal region of Tai. The presence of at least one equivalent indel-1 in Tai sequences of other insects, such as *T. castaneum*, suggests that this kind of Tai isoforms might be common in insects, and that this particular IN-1 can be of functional importance. Our observations that Tai isoforms are expressed with slightly different patterns during the last nymphal instars of *B. germanica* and at different levels depending on the tissue indicate that they may have specific functions. In the fat body, for example, isoforms A, C and D are well expressed, which can be related with the vitellogenic role of JH in this species [28], as occurs in *P. apterus*, where Tai is involved in the transduction of the JH vitellogenic signal [26]. In the ovary, the high expression of the C and D isoforms can be related with oogenesis processes, as occurs in *D. melanogaster* [16]; high expression of Tai in the ovaries has been also observed in *A. aegypti* [17]. The dramatically high expression of all BgTai isoforms in testes is interesting and merits a functional study.

### Tai and metamorphosis

Depletion of the four Tai isoforms (Tai-A to Tai-D) in *B. germanica* with effective doses of dsTai-core resulted in 100% mortality. However, we were able to determine the expression levels of the JH-dependent gene Kr-h1, which were lower than in controls, thus affording initial indications of possible functions of Tai in metamorphosis. Totally lethal effects were also observed after Tai depletion in larval stages of *T. castaneum* [25] and nymphs of *P. apterus* [26], which precluded any conclusion about Tai function in metamorphosis at the phenotypic level. In *T. castaneum*, Tai depletion in larvae affected lipid metabolism, which might contribute to the growth arrest and ultimately the mortality observed in these specimens [25].

When only the longer Tai-A and Tai-B isoforms were selectively and efficiently depleted in antepenultimate nymphal instar of *B. germanica*, mortality was only 18%, Kr-h1 expression was again reduced and, more interestingly, the experimental specimens underwent precocious morphogenesis of adult features after two moults (in what should be the last nymphal instar). These observations suggest that at least Tai-A or Tai-B or both are involved in transducing the JH signal that prevents metamorphosis in *B. germanica*. When the isoforms Tai-A and Tai-C were depleted, mortality was higher (48%), Kr-h1 levels were not affected, and survivors moulted to N6 and to adult normally. These negative results at phenotypic levels might be due to the modest levels of depletion reached by the dsRNA used, limited to a length of 74 nucleotides. Finally, when Tai-A, Tai-B and Tai-C were simultaneously, mortality was intermediate (35%), Kr-h1 mRNA levels tended to decrease (by 40%), but with no statistically significant differences in comparison with controls, and insects moulted to N5 normally and then to N6 that showed faint adult features (yellowish colouration and enlarged and flexible lateral expansions in T2 and T3).

It is difficult to interpret the combination of all results, especially because the RNAi of isoforms containing the IN-2 (Tai-A and Tai-C) was rather inefficient. The more robust results were obtained when depleting the isoforms containing the IN-1 (Tai-A and Tai-B), which showed that at least these isoforms are involved in transducing the anti-metamorphic signal of JH to Kr-h1. These results, however, do not rule out the possibility that the other isoforms are also involved in this role (as well as in other more vital functions), as our experiments cannot discard some degree of functional redundancy between all isoforms. Thus, the main conclusion derived from these RNAi studies *in vivo* is that one or more isoforms of Tai participate in the transduction of the JH signal that represses metamorphosis, which stands in itself as an innovative information.

According to the RNAi information, coupled with the available data *in vitro* that demonstrates the interaction of Met with Tai, and the functionality of the heterodimer Met-Tai as JH receptor [6], [18], [19], [23], we propose that one or more isoforms of Tai function as partners of Met in the reception of JH in metamorphosis repression in *B. germanica*. The JH signal would then be transduced to Kr-h1, which plays the role of adult morphogenesis master repressor, as its specific depletion triggers a clear-cut precocious metamorphosis, as evidenced elsewhere [22]. As there are no robust genetic information and tools in *B. germanica* to examine functional differences of different isoforms in the transduction of the JH signal to Kr-h1, we used *T. castaneum* for this purpose as TcTai sequences present an indel-1 (Figure S3) homologous to that of BgTai. Reporter assays indicated that both TcTai IN-1 and TcTai DEL-1 activated the kJHRE reporter when co-expressed with Met in the presence of JH. Intriguingly, TcTai DEL-1 was more efficient than TcTai IN-1, although the differences are minor and support the suggestion that all indel Tai isoforms may be involved in transducing the anti-metamorphic signal of JH to Kr-h1, although the biological context and the transduction efficiency can determine the use of either isoform.

### Tai versatility beyond metamorphosis

Moreover, Tai proteins can heterodimerise with Met and play roles in processes other than metamorphosis, like in the induction of vitellogenin transcription [26], [29]. They can bind to other partners, i.e., to other bHLH proteins or to nuclear receptors, like βFtz-F1 [17], thus contributing to modulate the expression of other genes in other pathways. Indeed, our results depleting BgTai indicate that in *B. germanica* it functions in the ecdysone signalling pathway, which has been previously reported in *D. melanogaster* [16] and *A. aegypti* [17], as well as in the insulin signalling pathway, a function that would deserve further research. Indeed, the information available indicates that Tai proteins may have different partners, which allows the possibility to perform a variety of tasks, and the data reported herein suggest that Tai isoforms may be influential in determining which partners are chosen and which roles are played. In mammals, SRC1 has at least 5 isoforms that differ in the C-terminal region [30] and which affect the interaction with nuclear receptors in different hormone signalling pathways [31]–[33]. In light of such versatile interaction capabilities and actions, vertebrate SRCs have been qualified, admittedly rather pompously, as masters of systems biology [34]. By extension, the Tai subfamily of SRC proteins may also play central roles in the circuitries regulating homeostasis and developmental processes in insects. Although this is a world yet to be explored, we predict that the Tai gene will fulfil a variety of roles in most cases taking advantage of the possibilities for versatility and promiscuity provided by the splicing isoforms.

## Materials and Methods

### Insects

Specimens of *B. germanica* were obtained from a colony reared in the dark at 30±1°C and 60–70% relative humidity. Freshly ecdysed female nymphs were selected and used at the appropriate ages. They were anaesthetized with carbon dioxide prior to injection treatments, dissections and tissue sampling.

### RNA extraction and retrotranscription to cDNA

We performed total RNA extraction from the whole body or specific tissues if indicated, using the miRNeasy extraction kit (QIAGEN). A 500-ng sample from each RNA extraction was treated with DNase (Promega) and reverse transcribed with first Strand cDNA Synthesis Kit (Roche) and random hexamers primers (Roche). RNA quantity and quality was estimated by spectrophotometric absorption at 260 nm using a Nanodrop Spectrophotometer ND-1000 (NanoDrop Technologies).

### Cloning and sequencing of BgTai and indel 1 fragment of TcTai cDNA

BgTai and ILP-1mRNAs were obtained from a *B. germanica* transcriptome prepared with RNA extracts from whole body of penultimate instar female nymphs and sequenced with a 454 Junior sequencer (Roche, Barcelona, Spain) at the Technical and Scientific Services of the Biomedical Research Park of Barcelona (PRBB). Transcriptome sequences were validated with RT-PCR using specific primers and cDNA from penultimate instar female nymphs of *B. germanica* as a template. Further 3′ and 5′ rapid amplification of cDNA ends (RACE, Ambion) allowed obtaining the complete ORF of BgTai and ILP-1. In the case of BgTai, 3′-RACE rendered four different isoforms based on two indels in the C-terminal region of the sequence. To amplify the fragment including the indel 1 of TcTai, we used primers based on the flanking motifs of this indel in the TcTai sequence (GenBank Accession number AB762694) (Table S2) and cDNA from last instar larvae of *T. castaneum* as a template. All PCR products were subcloned into the pSTBlue-1 vector (Novagen) and sequenced.

### Determination of mRNA levels by quantitative real-time PCR

Quantitative real time PCR (qRT-PCR) reactions were carried out in triplicate in an iQ5 Real-Time PCR Detection System (Bio-Rad Laboratories), using SYBR-Green (Power SYBR-Green PCR Master Mix; Applied Biosystems). A template-free control was included in all batches. The primers used to detect mRNA levels are described in Table S2. The efficiency of each set of primers was first validated by constructing a standard curve through four serial dilutions. Levels of mRNA were calculated relative to BgActin-5c (Accession number AJ862721) expression, using the Bio-Rad iQ5 Standard Edition Optical System Software (version 2.0). Results are given as copies of mRNA per 1000 copies of BgActin-5c mRNA or as standardized relative expression, setting controls levels to 1.0.

### Treatments *in vivo* with juvenile hormone III

Freshly emerged last instar female nymphs of *B. germanica* were topically applied in dorsal abdomen with JH III (Sigma-Aldrich), which is the native JH of B. *germanica* [35], at a dose of 20 µg diluted in 1 µL of acetone. Controls received 1 µL of acetone alone. The commercial JH III is a mixture of isomers containing *ca.* 50% of the biologically active (10*R*)-JH III, thus the active dose applied was around 10 µg per specimen.

### RNA interference

The detailed procedures for RNAi experiments were as described previously [36]. The different dsRNAs used for BgTai targeting as well as the primers to generate the corresponding templates are summarized in Table S2. The fragments were amplified by PCR and cloned into the pSTBlueTM-1 vector. A 307-bp sequence from *Autographa californica* nucleopoyhedrovirus was used as control dsRNA (dsMock). The dsRNAs were prepared as reported elsewhere [36]. A volume of 1 µL of dsRNA solution (3 µg/µL, unless stated otherwise) was injected into the abdomen of specimens at chosen ages and stages with a 5-µL Hamilton microsyringe. Control specimens were treated with the same dose and volume of dsMock.

### Micrographs and Scanning Electron Microscopy

Photomicrographs were taken under bright field using a Zeiss DiscoveryV8 Stereo microscope with an AxioCam MRc digital camera. For Scanning Electron Microscopy, samples were dried at room temperature for 2 weeks, posteriorly silver coated, and observed under a Hitachi S-57 scanning electron microscope.

### Reporter assays

Reporter plasmid carrying *k*JHRE and basal promoter of *TcKr-h1* (−477 to +1883) was used as described previously [24]. Expression plasmids of TcMet and TcTai DEL-1 for *Drosophila* S2 cells were constructed using the Gateway system (Invitrogen). The full ORFs of these cDNAs were amplified by PCR with Table S2 primer and pBIND_TcMet, pBIND_TcTai DEL-1 ( = TcSRC) [24] as the templates, and subcloned into pIZT vector (Invitrogen). To construct expression plasmid of TcTai IN-1, the IN-1 region was amplified by PCR using Table S2 primer and pSTBlue-1_TcTai IN-1 as a template, and pIZT_TcTai DEL-1 was amplified by inverse PCR using the KOD -Plus- Mutagenesis Kit (Toyobo) with Table S2 primer and pIZT_ TcTai DEL-1 as a template. The amplified IN-1 was inserted to pIZT_TcTai DEL-1 by blunt-end ligation (TOYOBO). S2 cells were seeded at a density of 2×10^5^ cells/well in 200 µl medium in a 96-well plate (Iwaki) one day before transfection. Transfections were performed by using the Fugene HD (Promega). The pIZT_RLuc vector containing the *Renilla* luciferase gene [37] was used as the reference. The cells were incubated for 48 h after transfection and treated with 1 µM of JH III for 24 h. They were then processed by using the Dual-Luciferase reporter assay system (Promega) in accordance with the manufacturer's instructions and analyzed with a luminometer (ARVO, PerkinElmer).

## Supporting Information

Figure S1 Phylogenetic analysis of insect Taiman/SRC/FISC proteins and vertebrate SRC proteins using maximum likelihood. The species and protein sequences included in the analysis were the following (the accession number is indicated in parenthesis; the protein name is the one used in the literature or in GenBank). Insects: *Acyrtosiphon pisum* SRC (XP_001944363); *Aedes aegypti* FISC (ABE99837); *Apis mellifera* NRC3 (XP_006563176); *Blattella germanica* Tai (CDO33883); *Bombyx mori* SRC (BAM17304); *Drosophila melanogaster* Tai (AAG16637); *Locusta migratoria* NcoA (AHA42532); *Pediculus humanus* hypothetical protein (assembly XP_002430185+XP_002430186); *Pyrrhocoris apterus* Tai/FISC (AGI17570); *Tribolium castaneum* SRC (XP_967666) and *Danaus plexippus* FISC (EHJ64466). Vertebrates: *Danio rerio* NRC 1 (XP_691744), NRC 2 (NP_571852) and NRC 3 (XP_692938); *Homo sapiens* SRC1 (NP_003734), SRC2 (NP_006531) and SRC3/AIB1 (AAC51677); *Mus musculus* SRC1 (XP_006515071), SRC2 (NP_032704) and SRC3 (NP_032705). The protein sequences were aligned using the MAFFT program (http://mafft.cbrc.jp/alignment/software), with the E-INS-I parameter. The model of protein evolution that best fits the data, determined using ProtTest 2.4 (http://darwin.uvigo.es/software/prottest2_server.html), was the LG+I+G+F, which was the one implemented in the maximum likelihood analyses. These were carried out with the PHYML version 3.0 program (http://www.atgc-montpellier.fr/phyml/). Data were bootstrapped for 100 replicates using the same program. Bootstrap values >50 are indicated on the corresponding node. The scale bar represents 0.3 substitutions per position.
(TIF)

Figure S2 Comparison of BgTai with other insect Tai and human SRC protein sequences. In addition to the bHLH region and PAS domains, the LxxLL motifs are also indicated (black bars). We indicate the percentage of overall identity and the percentage of identity for each of the characteristic domains of the protein. The species included are *Pediculus humanus* (Ph), *Locusta migratoria* (Lm), *Tribolium castaneum* (Tc), *Apis mellifera* (Am), *Pyrrhocoris apterus* (Pa), *Acyrtosiphon pisum* (Ap), *Aedes aegypti* (Aa), *Drosophila melanogaster* (Dm), *Homo sapiens* (Hs), *Danaus plexippus* (Dp) and *Bombyx mori* (Bm). The original protein names and the GenBank accession number of the sequences are indicated in Figure S1. * indicates that the sequence is incomplete in the region comprised between the initial Met and the bHLH domain and towards the C-terminal region.
(TIF)

Figure S3 Alignment of Taiman sequences of insect species having the indel-1 found in GenBank. We included the sequences of *Blattella germanica* (Bg) (with the insertion: CDO33883 and without the insertion CDO33885), *Tribolium. castaneum* (Tc) (with the insertion: XP_967666 and without the insertion: BAN62669), *Apis mellifera* (Am) (with the insertion: XP_006563176 and without the insertion: XP_006563185) and *Drosophila melanogaster* (Dm) (with the insertion, Tai-C: NP_001188746 and without the insertion, Tai-F: NP_001188748). The protein sequences were aligned using the MAFFT algorithm following the procedure described in Figure S1 and visualized in Geneioius Software. Positions with 100% of identity are indicated in black, 80 to 99% in dark grey, 60 to 79% in bright grey and in white with less than 59%. The blue and red square frame the insertion and the deletion, respectively.
(TIF)

Figure S4 Sequences obtained after amplifying the region around the indel-1 in *Tribolium castaneum* Tai (TcTai) using the specific primers described in Table S2, compared with the equivalent sequences of *Blattella germanica* Taiman (BgTai). Two types of amplicons were obtained, one showing the insertion-1 (TcTai-IN-1), and the other without it (TcTai-DEL-1). Alignment and visualization was carried out as in Figure S3. The blue square frames the stretch of amino acids corresponding to the indel-1.
(TIF)

Figure S5 Phenotypes obtained after depleting the ensemble of *Blattella germanica* Tai isoforms with dsTai-core. (A) Dorsal and ventral view of a specimen that had been treated with high doses of dsTai-core (2 doses of 3 µg each, one on N5D0 and the other on N5D3) and photographed on N5D11; in general, these specimens showed reduced motility, stopped growing, became darker and finally died 7–10 days after the administration of the second dose on N5D3. (B) Dorsal and ventral view of a specimen that had been treated with a low dose of dsTai-core (1 dose of 0.2 µg on N5D0) and photographed on N6; this specimen shows the lateral expansions of T2 and T3 slightly longer and apparently more transparent than controls (indicated with an arrow in the detail), although it subsequently moulted to a normal adult.
(TIF)

Figure S6 Wing morphology just before the imaginal ecdysis in *Blattella germanica*. (A) Normal N6 female on day 7, thus just before starting the ecdysis. (B) Detail of the right lateral expansion of T2 forming a pocket that contains the developing wing (tegmina). (C) Heavily folded developing wing (tegmina) dissected out from the lateral T2 pocket.
(TIF)

Table S1 Summary of the effects of Tai depletion at phenotypic level in the experiments treating with dsTai-core, dsTai-in-1 and dsTai-in-2 at the instar N4 and/or N5. The corresponding dsRNA was administered in one or two doses depending on the experiment. Controls were equivalently treated with dsMock as indicated. Methodological details are described in the main text.
(PDF)

Table S2 Primers used to detect transcript levels by qPCR in *Blattella germanica* tissues, to amplify the indel-1 region of *Tribolium castaneum* Taiman, to prepare the dsRNAs for RNAi experiments, and to amplify the full ORF of TcTai IN-1 and TcTai DEL-1 from *T. castaneum* tissues.
(PDF)
