# Supplementary figures and images for: A Role for Taiman in Insect Metamorphosis
Source: PLoS Genet. 2014 Oct 30;10(10):e1004769. doi: 10.1371/journal.pgen.1004769 (PMC4214675; doi:10.1371/journal.pgen.1004769)

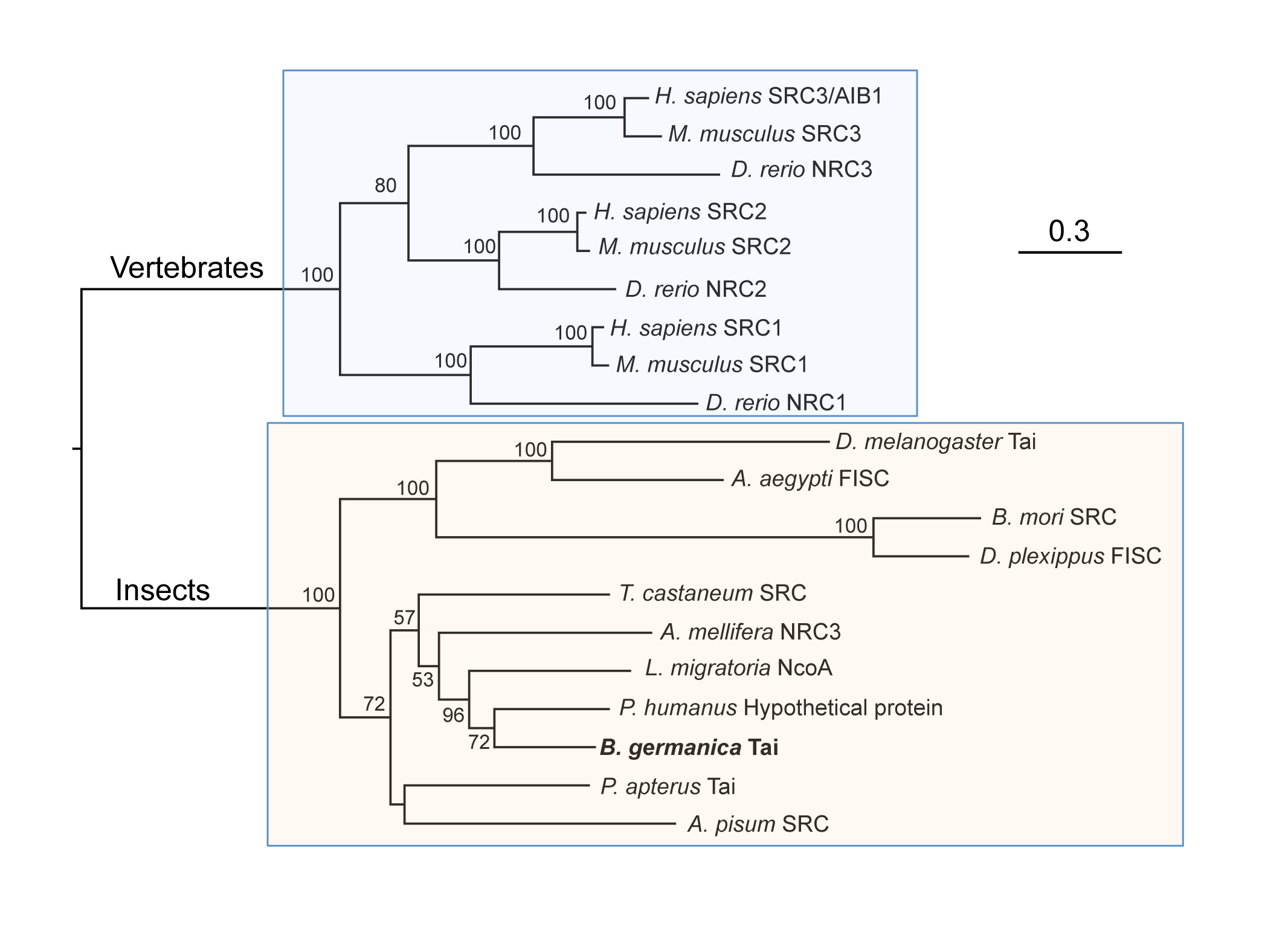

Supplement: Figure S1 — Phylogenetic analysis of insect Taiman/SRC/FISC proteins and vertebrate SRC proteins using maximum likelihood. The species and protein sequences included in the analysis were the following (the accession number is indicated in parenthesis; the protein name is the one used in the literature or in GenBank). Insects: Acyrtosiphon pisum SRC (XP_001944363); Aedes aegypti FISC (ABE99837); Apis mellifera NRC3 (XP_006563176); Blattella germanica Tai (CDO33883); Bombyx mori SRC (BAM17304); Drosophila melanogaster Tai (AAG16637); Locusta migratoria NcoA (AHA42532); Pediculus humanus hypothetical protein (assembly XP_002430185+XP_002430186); Pyrrhocoris apterus Tai/FISC (AGI17570); Tribolium castaneum SRC (XP_967666) and Danaus plexippus FISC (EHJ64466). Vertebrates: Danio rerio NRC 1 (XP_691744), NRC 2 (NP_571852) and NRC 3 (XP_692938); Homo sapiens SRC1 (NP_003734), SRC2 (NP_006531) and SRC3/AIB1 (AAC51677); Mus musculus SRC1 (XP_006515071), SRC2 (NP_032704) and SRC3 (NP_032705). The protein sequences were aligned using the MAFFT program (http://mafft.cbrc.jp/alignment/software), with the E-INS-I parameter. The model of protein evolution that best fits the data, determined using ProtTest 2.4 (http://darwin.uvigo.es/software/prottest2_server.html), was the LG+I+G+F, which was the one implemented in the maximum likelihood analyses. These were carried out with the PHYML version 3.0 program (http://www.atgc-montpellier.fr/phyml/). Data were bootstrapped for 100 replicates using the same program. Bootstrap values >50 are indicated on the corresponding node. The scale bar represents 0.3 substitutions per position. (TIF) [file pgen.1004769.s001.tif]

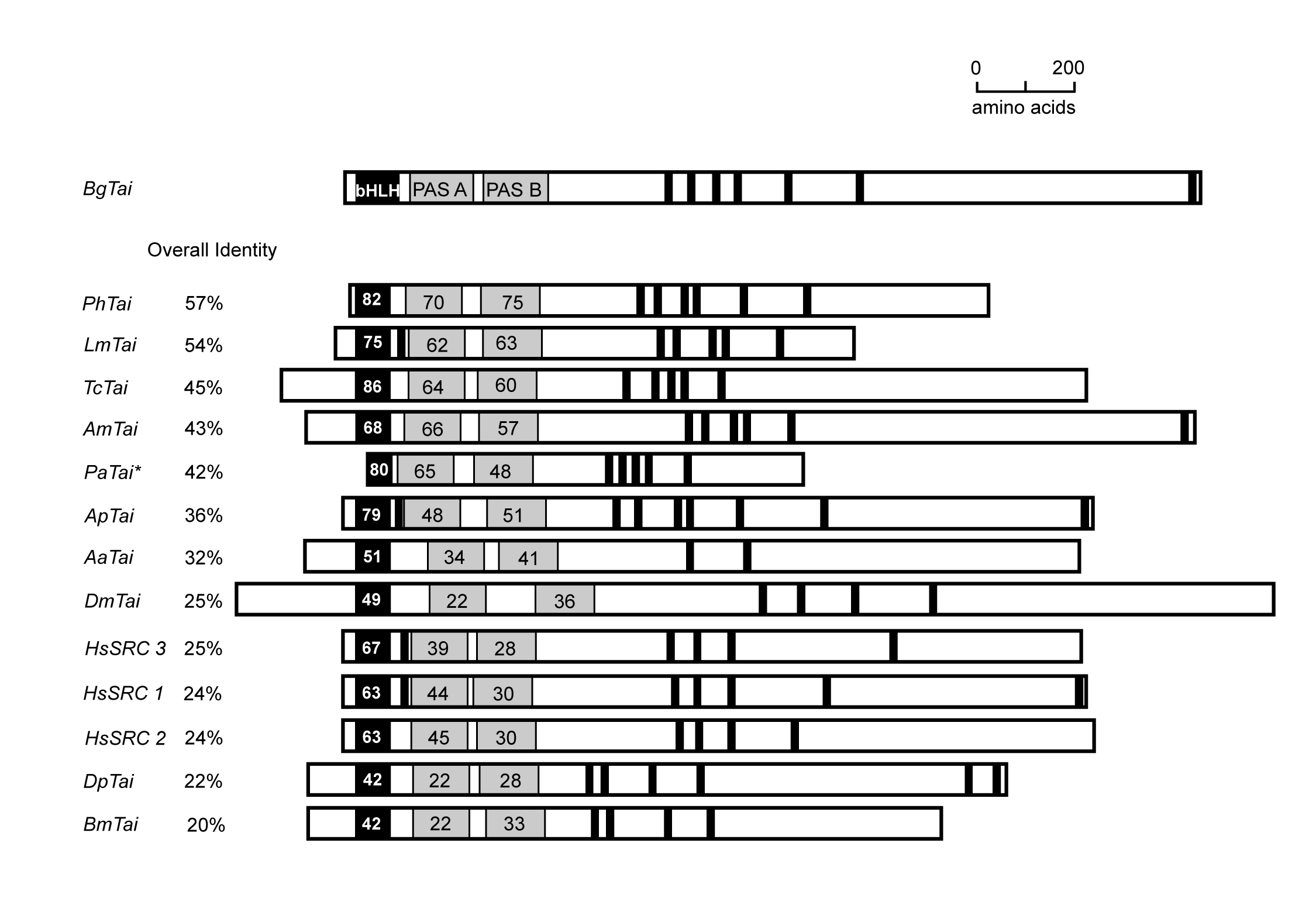

Supplement: Figure S2 — Comparison of BgTai with other insect Tai and human SRC protein sequences. In addition to the bHLH region and PAS domains, the LxxLL motifs are also indicated (black bars). We indicate the percentage of overall identity and the percentage of identity for each of the characteristic domains of the protein. The species included are Pediculus humanus (Ph), Locusta migratoria (Lm), Tribolium castaneum (Tc), Apis mellifera (Am), Pyrrhocoris apterus (Pa), Acyrtosiphon pisum (Ap), Aedes aegypti (Aa), Drosophila melanogaster (Dm), Homo sapiens (Hs), Danaus plexippus (Dp) and Bombyx mori (Bm). The original protein names and the GenBank accession number of the sequences are indicated in Figure S1. * indicates that the sequence is incomplete in the region comprised between the initial Met and the bHLH domain and towards the C-terminal region. (TIF) [file pgen.1004769.s002.tif]

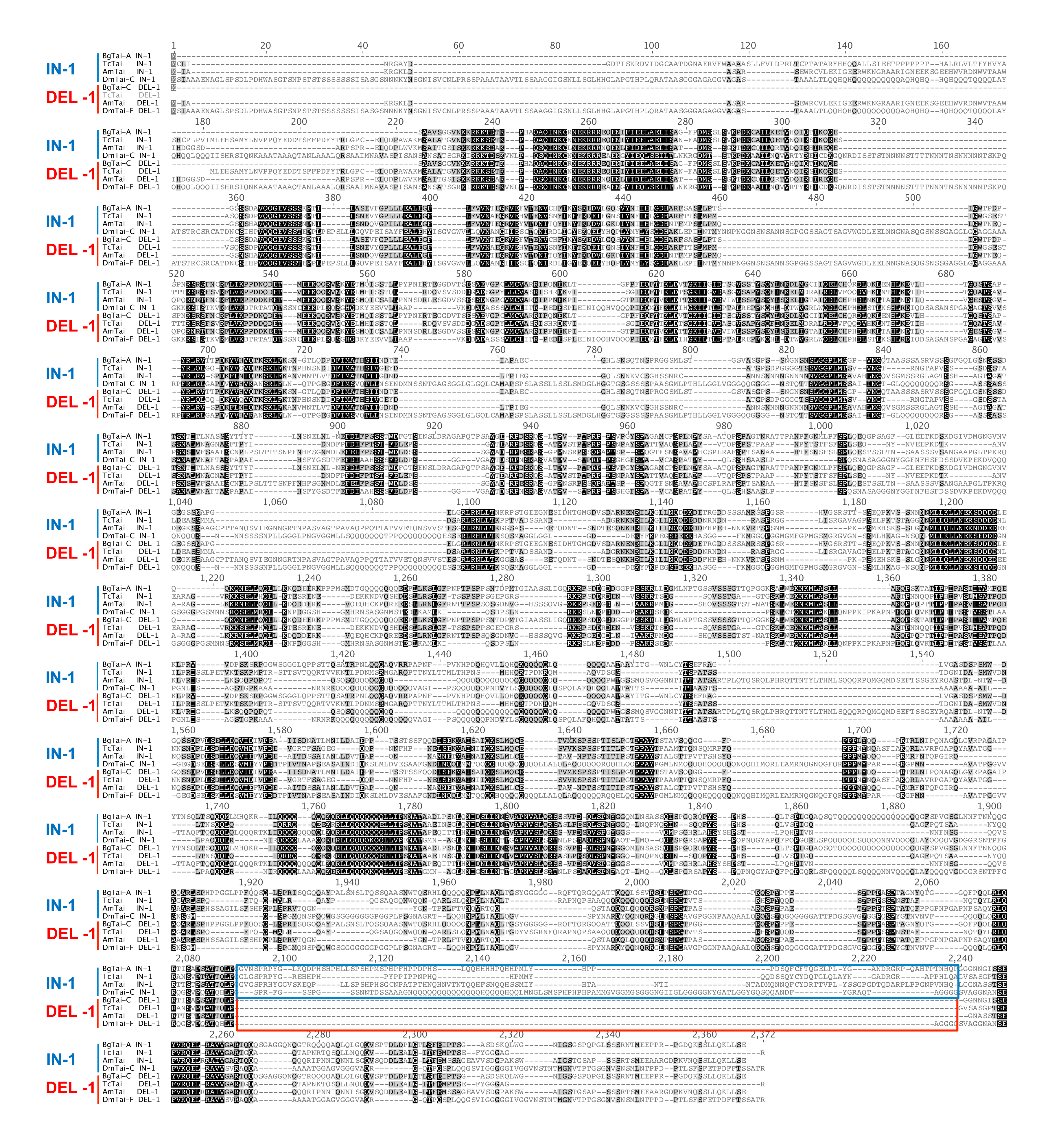

Supplement: Figure S3 — Alignment of Taiman sequences of insect species having the indel-1 found in GenBank. We included the sequences of Blattella germanica (Bg) (with the insertion: CDO33883 and without the insertion CDO33885), Tribolium. castaneum (Tc) (with the insertion: XP_967666 and without the insertion: BAN62669), Apis mellifera (Am) (with the insertion: XP_006563176 and without the insertion: XP_006563185) and Drosophila melanogaster (Dm) (with the insertion, Tai-C: NP_001188746 and without the insertion, Tai-F: NP_001188748). The protein sequences were aligned using the MAFFT algorithm following the procedure described in Figure S1 and visualized in Geneioius Software. Positions with 100% of identity are indicated in black, 80 to 99% in dark grey, 60 to 79% in bright grey and in white with less than 59%. The blue and red square frame the insertion and the deletion, respectively. (TIF) [file pgen.1004769.s003.tif]

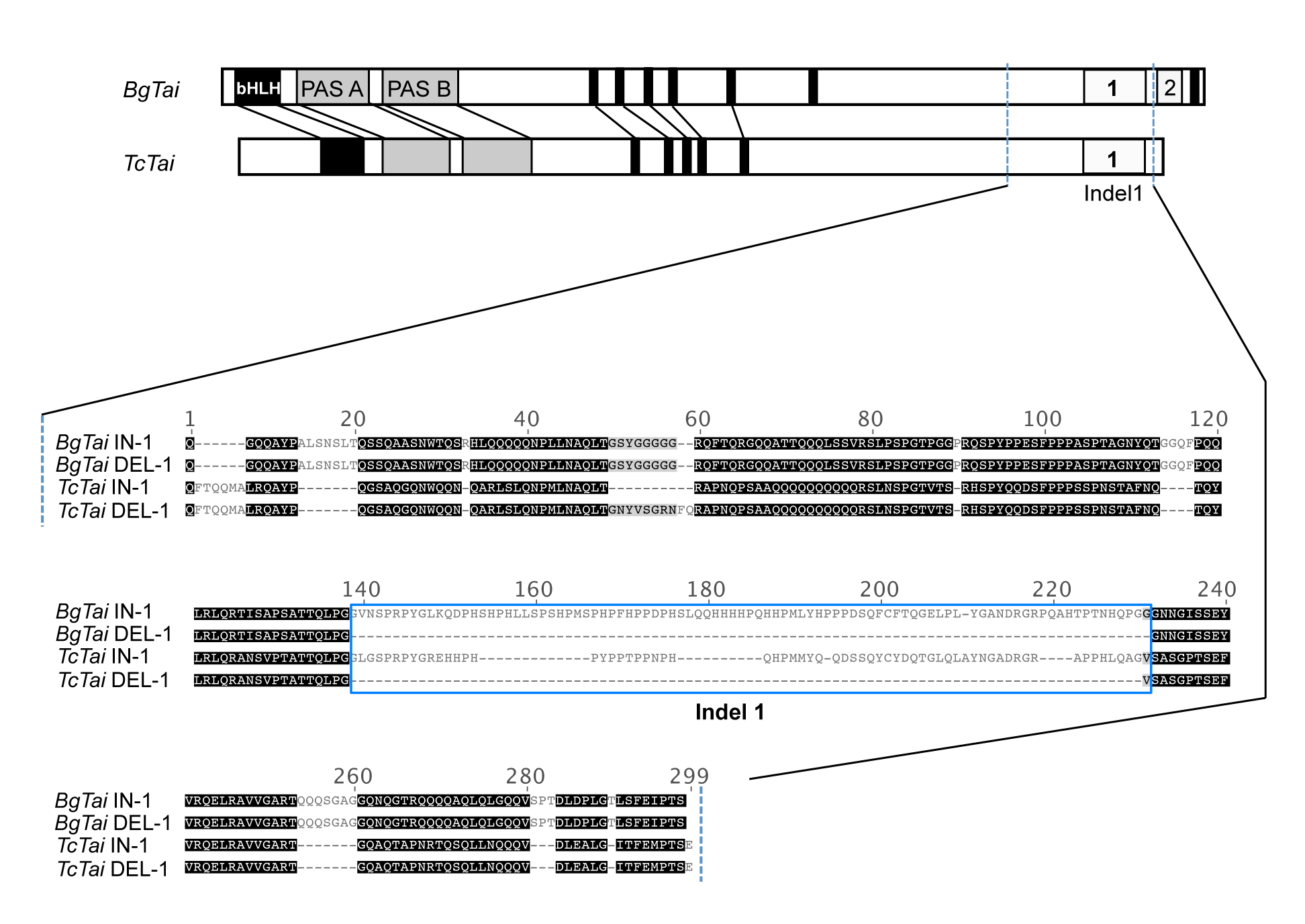

Supplement: Figure S4 — Sequences obtained after amplifying the region around the indel-1 in Tribolium castaneum Tai (TcTai) using the specific primers described in Table S2, compared with the equivalent sequences of Blattella germanica Taiman (BgTai). Two types of amplicons were obtained, one showing the insertion-1 (TcTai-IN-1), and the other without it (TcTai-DEL-1). Alignment and visualization was carried out as in Figure S3. The blue square frames the stretch of amino acids corresponding to the indel-1. (TIF) [file pgen.1004769.s004.tif]

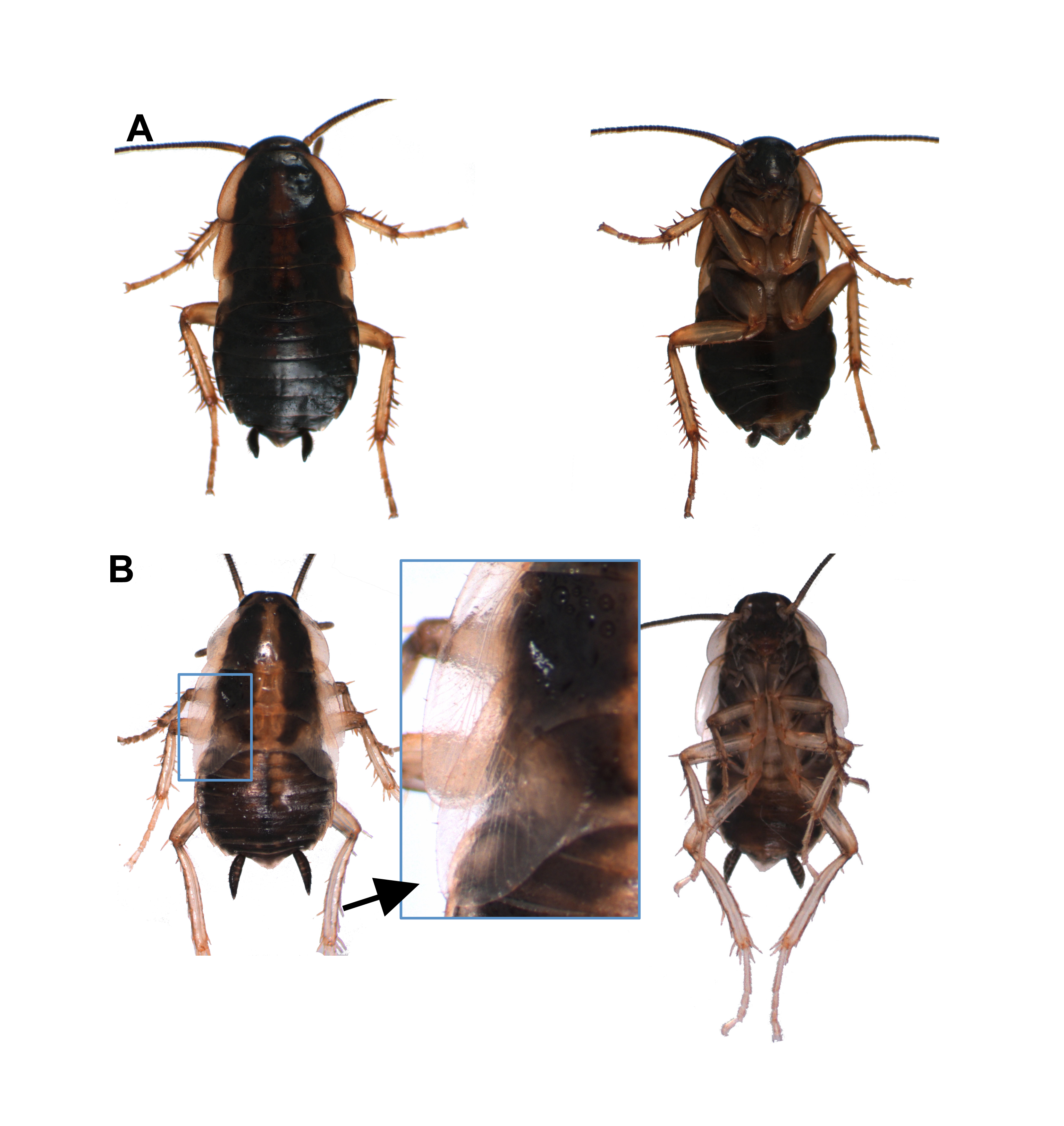

Supplement: Figure S5 — Phenotypes obtained after depleting the ensemble of Blattella germanica Tai isoforms with dsTai-core. (A) Dorsal and ventral view of a specimen that had been treated with high doses of dsTai-core (2 doses of 3 µg each, one on N5D0 and the other on N5D3) and photographed on N5D11; in general, these specimens showed reduced motility, stopped growing, became darker and finally died 7–10 days after the administration of the second dose on N5D3. (B) Dorsal and ventral view of a specimen that had been treated with a low dose of dsTai-core (1 dose of 0.2 µg on N5D0) and photographed on N6; this specimen shows the lateral expansions of T2 and T3 slightly longer and apparently more transparent than controls (indicated with an arrow in the detail), although it subsequently moulted to a normal adult. (TIF) [file pgen.1004769.s005.tif]

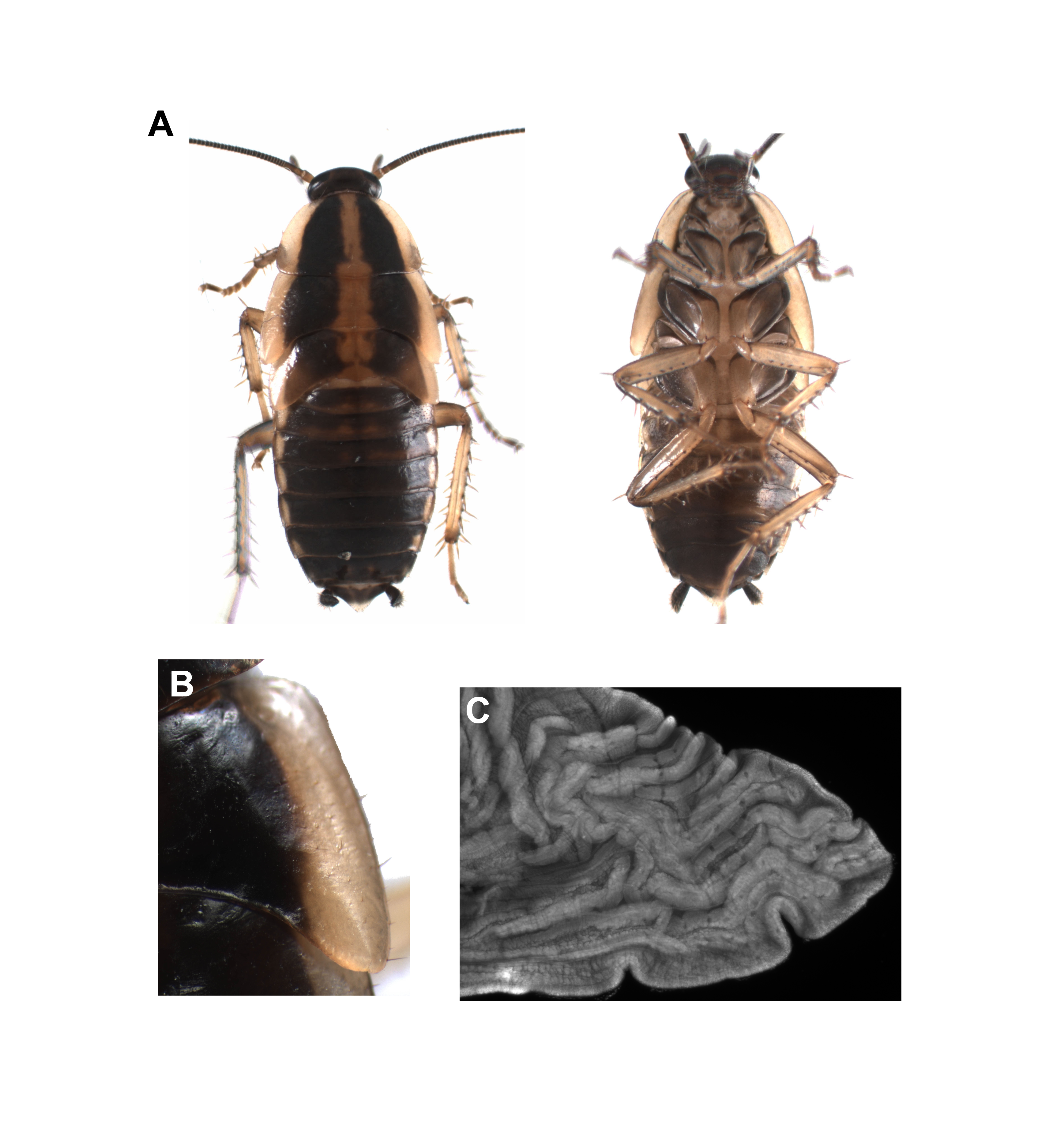

Supplement: Figure S6 — Wing morphology just before the imaginal ecdysis in Blattella germanica. (A) Normal N6 female on day 7, thus just before starting the ecdysis. (B) Detail of the right lateral expansion of T2 forming a pocket that contains the developing wing (tegmina). (C) Heavily folded developing wing (tegmina) dissected out from the lateral T2 pocket. (TIF) [file pgen.1004769.s006.tif]
