# Supplementary material for: A Role for Taiman in Insect Metamorphosis
Source: PLoS Genet. 2014 Oct 30;10(10):e1004769. doi: 10.1371/journal.pgen.1004769 (PMC4214675; doi:10.1371/journal.pgen.1004769)
Supplement: Table S1 — Summary of the effects of Tai depletion at phenotypic level in the experiments treating with dsTai-core, dsTai-in-1 and dsTai-in-2 at the instar N4 and/or N5. The corresponding dsRNA was administered in one or two doses depending on the experiment. Controls were equivalently treated with dsMock as indicated. Methodological details are described in the main text. (PDF) [file pgen.1004769.s007.pdf]

Table S1. Summary of the effects of Tai depletion at phenotypic level in the experiments treating with dsTai-core, dsTai-in-1 and dsTai-in-2 at the instar N4 and/or N5. The corresponding dsRNA was administered in one or two doses depending on the experiment. Controls were equivalently treated with dsMock as indicated. Methodological details are described in the main text.

Instar of treatment	Dose applied each day	dsRNA day 0	dsRNA day 3	N	Died before N6	Died N6 and Adult	**Nymphoid with adult features in N6**	**Precocious adult in N6**	Adult with stretched wings after N6	Normal adult after N6
N5	3 µg	dsTai-core	dsTai-core	18	18	0	**0**	**0**	0	0
N5	3 µg	dsMock	dsMock	14	0	0	**0**	**0**	0	14
N5	0.5 µg	dsTai-core	-	16	14	0	**0**	**0**	1	1
N4	3 µg	dsTai-core	dsTai-core	12	12	0	**0**	**0**	0	0
N4	3 µg	dsMock	dsMock	15	0	0	**0**	**0**	0	15
N4	0.5 µg	dsTai-core	-	7	7	0	**0**	**0**	0	0
N4	0.3 µg	dsTai-core	-	16	6	0	**0**	**0**	6	4
N4	0.2 µg	dsTai-core	-	12	0	0	**0**	**0**	8	4
N4	3 µg	dsTai-in-1	dsTai-in-1	27	5	0	**19**	**3**	0	0
N4	3 µg	dsTai-in-2	dsTai-in-2	33	8	8	**0**	**0**	0	17
N4	3 µg	dsMock	dsMock	10	0	0	**0**	**0**	0	10
N4	3 + 3 µg	dsTai-in-1 + dsTai-in-2	dsTai-in-1 + dsTai-in-2	20	7	0	**13**	**0**	0	0
N4	6 µg	dsMock	dsMock	15	0	0	**0**	**0**	0	15
