# Supplementary material for: A Role for Taiman in Insect Metamorphosis
Source: PLoS Genet. 2014 Oct 30;10(10):e1004769. doi: 10.1371/journal.pgen.1004769 (PMC4214675; doi:10.1371/journal.pgen.1004769)
Supplement: Table S2 — Primers used to detect transcript levels by qPCR in Blattella germanica tissues, to amplify the indel-1 region of Tribolium castaneum Taiman, to prepare the dsRNAs for RNAi experiments, and to amplify the full ORF of TcTai IN-1 and TcTai DEL-1 from T. castaneum tissues. (PDF) [file pgen.1004769.s008.pdf]

Table S2. Primers used to detect transcript levels by qPCR in *Blattella germanica* tissues, to amplify the indel-1 region of *Tribolium castaneum* Taiman (TcTai indel 1), to prepare the dsRNAs for RNAi experiments and to construct the expression plasmids for reporter assays.

Primer set	Length (bp)	Forward primer (5'-3')	Reverse primer (5'-3')	Encompassed Region
Met	58 bp	CTGTTGGGACATCAGCAGAA	GGCAGGTGATGGAGTGAAGT	Nucleotide 470 to 527 of HG965209
Kr-h1	77 bp	GCGAGTATTGCAGCAAATCA	GGGACGTTCTTTCGTATGGA	Nucleotide 493 to 569 of HE575250
BR-C	76 pb	CGGGTCGAAGGGAAAGACA	CTTGGCGCCGAATGCTGCGAT	Nucleotide 699 to 774 of FN651774
Tai	50 pb	GCAGCGAGTAATTGGACACA	TTTTGCTGTTGCTGTTGGAG	Nucleotide 4288 to 4337 of HG965205
EcR	163 bp	GACAAACTCCTCAGAGAAGATCAA A	CTCCCAATCCTGCCAGACTA	Nucleotide 1472 to 1634 of AM039690
RXR	86 pb	ATAATTGACAAGAGGCAGAGGAA	TGAACAGCCTCCCTCTTCAT	Nucleotide 527 to 612 of AJ854490
E75A	101 bp	GTGCTATTGAGTGTGCGACATGAT	TCATGATCCCTGGAGTGGTAGAT	Nucleotide 58 to 158 of AM238653
ILP-1	96 bp	AGAAGCAGAATTCCCTTTCCG	TCATCGACAATGCCTCCGT	Nucleotide 343 to 438 of BgILP-1 HG972850
Tai-A	260 bp	CACACACCAACCAATCACCA	CCCCAAAGTTGCTTGCTATC	Nucleotide 4870 to 5129 of HG965205
Tai-B	241 bp	CACACACCAACCAATCACCA	CATAGTATTCAGAGGTAGGAA	Nucelotide 4870 to 5110 of HG965206
Tai-C	241 bp	AGTTGCCAGGTGGTAACAATG	CCCCAAAGTTGCTTGCTATC	Nucleotide 4613 to 4852 of HG965207
Tai-D	222 pb	AGTTGCCAGGTGGTAACAATG	CATAGTATTCAGAGGTAGGAA	Nucleotide 4613 to 4834 of HG965208
Actin	213 pb	AGCTTCCTGATGGTCAGGTGA	TGTCGGCAATTCCAGGGTACATGG T	Nucleotide 96 to 308 of AJ862721
dsTai-core	343 bp	CAACTTCAACAGCAGCAGCAA	AAGGACTCTTCATTACTGTT	Nucleotide 3169 to 3511 of HG965205
dsTai-IN-1	276 pb	TGGAGTAAACTCCCCGCGGC	CCACCGGGCTGGTGATTGGT	Nucleotide 4623 to 4789 of HG965205
dsTai-IN-2	74 pb	GGTGCCAGTGATAGCAAGCA	CTTGAGGAGGAGAGCCCGGG	Nucleotide 5101 to 5174 of HG965205
dsMock	307 bp	ATCCTTTCCTGGGACCCGGCA	ATGAAGGCTCGACGATCCTA	Nucleotide 370 to 676 of K01149
TcTai indel 1	551 bp	ACAGTTCACGCAACAGATGG	AACTCAGACGTGGGCATTTC	Nucleotide 3777 to 4490 of XM_962573
pIZT_TcMet	1578 bp	AAAAAGCAGGCTTCGAAGGAGATA GAACCATGGTAGCTCCTTGTGAAC TC	AGAAAGCTGGGTCTCATACTTTGT TACGAAGTAGTTGG	Nucleotide 139 to 1716 of AB360765
pIZT_TcTai DEL-1	4056 bp	AAAAAGCAGGCTTCGAAGGAGATA GAACCATGTTGGAACACAGTGCTA TGTATT	AGAAAGCTGGGTCTCACCTGCCC GCGCCTC	Nucleotide 1 to 4056 of AB762694
Insert for pIZT_TcTai IN-1	192 bp	GGCCTGGGATCCCCCCGGC	ACCGCCTTGGAGGTGTGGGGG	---
Vector for pIZT_TcTai IN-1	7461 bp	GTGTCCGCAAGTGGCCCCACATC	ACCTGGCAACTGGGTTGTGGCAG TG	pIZT_TcTai DEL-1 region distributed between 3855 and 3856 of TcTai DEL-1
